# Autophagic Flux Unleashes GATA4-NF-*κ*B Axis to Promote Antioxidant Defense-Dependent Survival of Colorectal Cancer Cells under Chronic Acidosis

**DOI:** 10.1155/2021/8189485

**Published:** 2021-12-26

**Authors:** Xiaojie Liu, Minnan Zhao, Xue Sun, Zhenzhen Meng, Xiaojing Bai, Yanchao Gong, Limei Xu, Xiaohe Hao, Tingting Yang, Zhao Wei, Xiyu Zhang, Haiyang Guo, Peishan Li, Qiao Liu, Yaoqin Gong, Yufang Shi, Changshun Shao

**Affiliations:** ^1^The Third Affiliated of Soochow University, State Key Laboratory of Radiation Medicine and Protection, Institutes for Translational Medicine, Soochow University Medical College, Suzhou, Jiangsu 215123, China; ^2^The Affiliated Hospital of Qingdao University, Qingdao, Shandong 266000, China; ^3^Key Laboratory of Experimental Teratology, Ministry of Education/Department of Molecular Medicine and Genetics, Shandong University School of Medicine, Jinan, Shandong 250012, China; ^4^Department of Clinical Laboratory, Qilu Hospital, Shandong University, Jinan, Shandong 250012, China; ^5^The Second Hospital of Shandong University, Jinan, Shandong 250033, China

## Abstract

Solid tumors are usually associated with extracellular acidosis due to their increased dependence on glycolysis and poor vascularization. Cancer cells gradually become adapted to acidic microenvironment and even acquire increased aggressiveness. They are resistant to apoptosis but exhibit increased autophagy that is essential for their survival. We here show that NF-*κ*B, a master regulator of cellular responses to stress, is upregulated in colorectal cancer cells adapted to acidosis (CRC-AA). NF-*κ*B is more relied upon for survival in CRC-AA than in their parental cells and drives a robust antioxidant response. Supplementation of antioxidant abolishes the increased sensitivity of CRC-AA to NF-*κ*B inhibition or depletion, suggesting that NF-*κ*B supports the survival of CRC-AA by maintaining redox homeostasis. Because SQSTM1/p62 is known to mediate the selective autophagy of GATA4 that augments NF-*κ*B function, we tested whether the enhanced autophagic flux and consequently the reduction of SQSTM1/p62 in CRC-AA cells could activate the GATA4-NF-*κ*B axis. Indeed, GATA4 is upregulated in CRC-AA cells and augments the NF-*κ*B activity that underlies the increased expression of cytokines, inhibition of apoptosis, and reduction of reactive oxygen species. Interestingly, secretory factors derived from HCT15-AA cells, the soluble ICAM-1 in particular, also possess antioxidant cytoprotective effect against acidic stress. Together, our results demonstrate a prosurvival role of the p62-restricted GATA4-NF-*κ*B axis in cancer cells adapted to acidic microenvironment.

## 1. Introduction

Cancer cells reside in a complex milieu or microenvironment that comprises stromal cells, immune cells, extracellular matrices, cytokines, and metabolites. Tumor progression is strongly influenced by the properties of the tumor microenvironment [1, 2]. Tumor microenvironment is usually hypoxic, poorly vascularized, and low in nutrients [2–4]. Notably, tumor cells remain highly glycolytic even in oxygenated environments and thus produce a lot of lactic acid. Due to the high rate of glycolysis in cancer cells and the poor vascularization in cancer tissues, cancer cells are usually immersed in an acidic microenvironment, which is also known as extracellular acidosis [5–7]. While acute acidic exposure can be cytotoxic, tumor cells always become adapted to a chronic extracellular acidosis [2, 8–11]. They even exhibit a higher intracellular pH (pH > 7.4), which is regarded as one of the adaptive features in most cancers [2, 12].

As a cellular catabolic pathway, autophagy plays a critical role for the survival of cells under various types of stress [13–15]. Nutritional deprivation, high temperature, infection, extracellular acidosis, and hypoxia are all known to increase the level of ROS and induce autophagy [16–18]. Even cells under normal physiological conditions rely on moderate levels of autophagic activity to preserve cellular homeostasis [19]. Several studies show that autophagy is critical for the survival of cancer cells in an acidic microenvironment [20–23]. Autophagy was elevated in MDA-MB-231 breast cancer cells after their exposure to low pH (6.7). Melanoma cells promptly accumulated LC3^+^ autophagic vesicles after exposing to acidic culture conditions (7.0 < pH < 6.2). These studies indicate that increased autophagy represents an essential adaptation mechanism for cancer cells exposed to acidic stress. We previously showed that while colorectal cancer (CRC) cells exhibited an increased level of reactive oxygen species (ROS) when acutely exposed to acidic medium, the CRC cells that eventually became adapted to low extracellular pH, after being passaged in acidic medium (pH 6.5) continuously for at least three months, had reduced level of ROS and were more sensitive to glutathione-depleting agent than their parental cells [24]. However, how the CRC cells that became acclimated to the acidic microenvironment, designated as CRC-AA, acquire the increased antioxidant capacity remains to be elucidated. In this study, we further characterized the CRC-AA cells in terms of their dependence on autophagy and explored the molecular underpinnings that confer CRC-AA cells the increased antioxidant function. As expected, autophagic flux is increased in the CRC-AA cells and is essential for their survival. We found that NF-*κ*B was upregulated and was more relied upon in CRC-AA than in their parental cells. Importantly, the NF-*κ*B upregulation was driven by an enhanced accumulation of GATA4, which escaped from p62-mediated selective degradation. The GATA4-NF-*κ*B pathway boosted antioxidant defense and thus enabled the CRC-AA cells to survive under the low extracellular pH.

## 2. Results

### 2.1. Autophagy Promotes Survival of Colorectal Cancer Cells under Acidic Microenvironment

**Figure 1:**
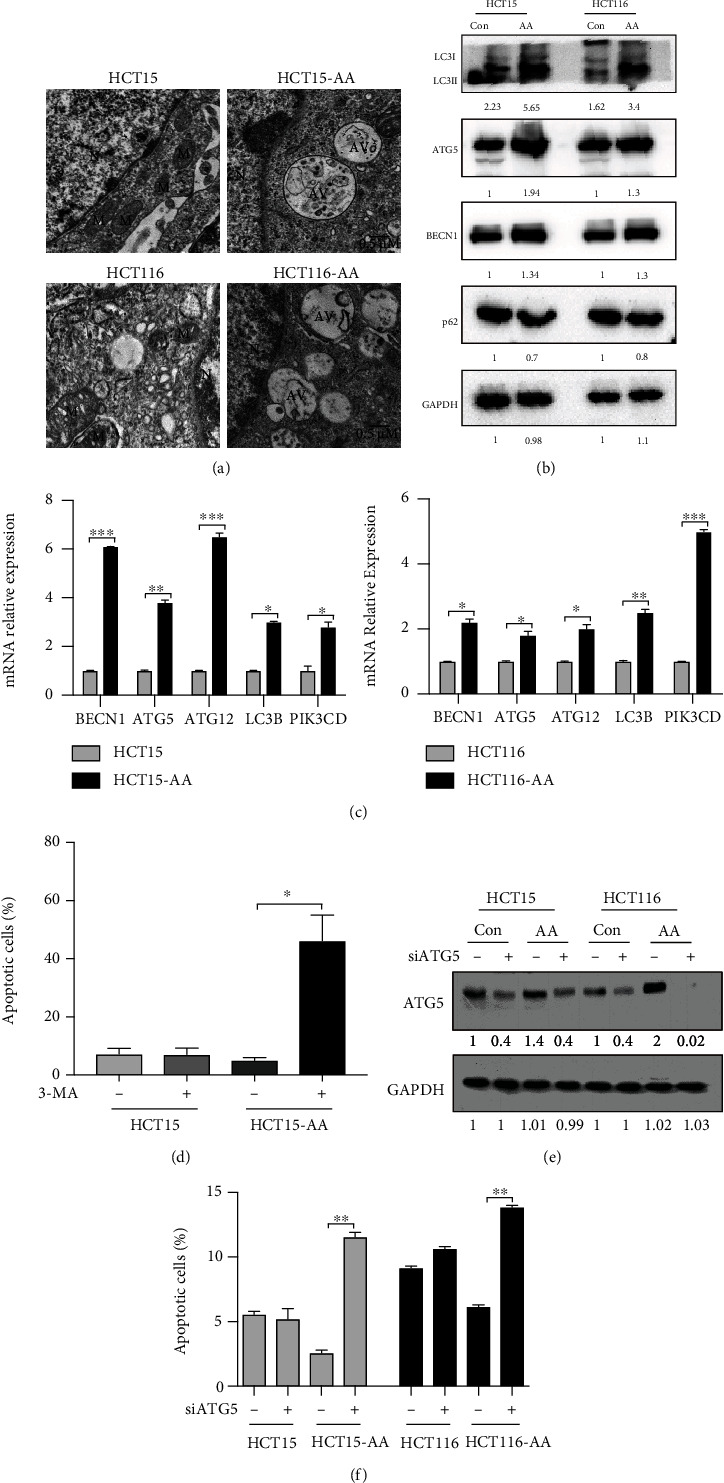
Autophagy promotes survival of colorectal cancer cells under acidic microenvironment. CRC and CRC-AA cells were cultured in pH 7.4 and pH 6.5, respectively. (a) Autophagy in CRC and CRC-AA cells examined with TEM (AV: autophagic vacuole; M: mitochondria; N: nucleus). (b) Expression of autophagy-related genes measured with Western blotting analysis. (c) mRNA levels of autophagy-related genes measured by qPCR. (d) Apoptotic levels of HCT15 and HCT15-AA cells after treatment with 3-MA (5 mM/L) for 24 h. (e) Depletion of ATG5 by siRNA in CRC-AA and their parental (HCT15, HCT116) cells. RNAi efficiency was determined by Western blotting analysis 48 h after transfection. GAPDH was used as a loading control. (f) Percentages of apoptotic cells in CRC and CRC-AA cells under the indicated conditions. The data shown were representative of three independent experiments. ^∗^*p* < 0.05; ^∗∗^*p* < 0.01; ^∗∗∗^*p* < 0.001.

Autophagy was a protective cellular response to adversity. To assess the level of autophagy in the CRC-AA cells and test whether autophagy is critically required for the survival of the cancer cells under low extracellular pH, we first examined autophagic vacuoles by transmission electron microscopy (TEM). As shown in Figure 1(a), CRC-AA cells had more vacuolar structures composed of two-layer membranes, in which organelles were encapsulated, indicative of increased autophagy in CRC-AA cells when compared to CRC cells. Moreover, autophagy-related genes, such as BECN1, ATG5, and LC3B, were significantly upregulated in CRC-AA cells (Figures 1(b) and 1(c)). CRC-AA cells also exhibited a higher LC3BII/LC3BI ratio and a lower p62 (SQSTM1) level than their parental cells (Figure 1(b)), suggesting that there is a more robust autophagic flux in CRC-AA cells than in the parental CRC cells. As a control, when autophagic flux was blocked using ammonium chloride, a lysosomotropic agent that raises intralysosomal pH, the ratio of LC3-II/LC3-I and the p62 protein level were increased in CRC and CRC-AA cells (Figure S1a). As a mechanism for maintaining cellular homeostasis and survival, autophagy is expected to alleviate endoplasmic reticulum (ER) stress [25]. We examined three markers of ER stress, ATF4, CHOP, and p-eIF2*α*, and found that they were all reduced in CRC-AA cells when compared to the parental CRC cells (Figure S2a).

To evaluate the role of autophagy for the survival of CRC-AA cells, we treated parental and CRC-AA cells with 6-amino-3-methylindole (3-MA), an autophagy inhibitor, for 24 h. As shown in Figure 1(d), suppression of autophagy significantly increased the proportion of apoptotic cells compared with parental cells. Consistently, when ATG5 was knocked down by RNAi (Figure 1(e)), apoptosis was significantly elevated in CRC-AA cells, but remained unchanged in CRC cells (Figure 1(f)). These results suggest that CRC-AA cells are more reliant on autophagy for survival than their parental cells.

### 2.2. NF-*κ*B Is Upregulated in CRC-AA Cells

**Figure 2:**
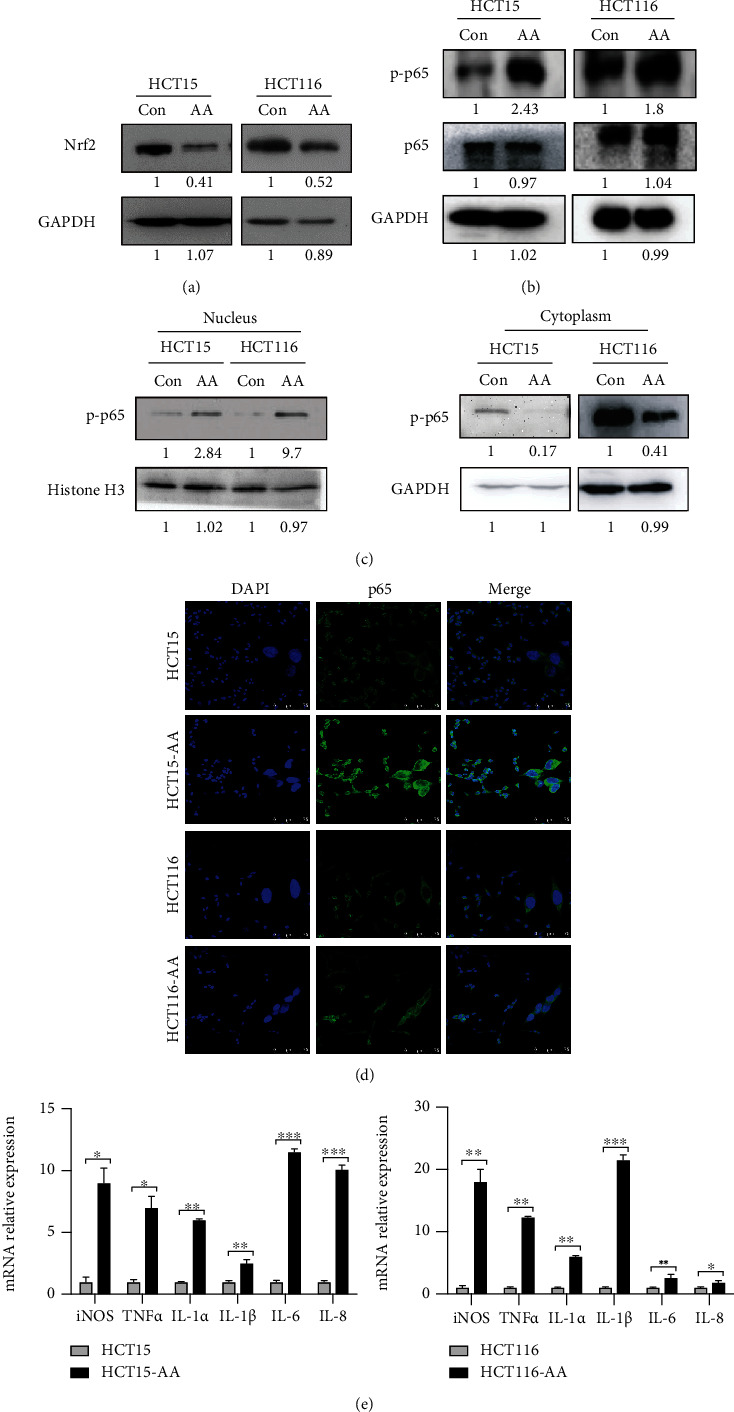
NF-*κ*B is upregulated in CRC-AA cells. (a) The expression of NRF2 in CRC-AA and their parental cells was measured by Western blotting. (b) Levels of p-p65 and p65 determined by Western blotting. (c) Western blot analysis of p-p65 in cytoplasmic and nucleus components, respectively. GAPDH and histone H3 were used as loading controls for the cytoplasmic and nuclear proteins, respectively. (d) Immunofluorescence staining of NF-*κ*B/p65 in CRC and CRC-AA cells. (e) mRNA levels of cytokines measured by quantitative real-time PCR. Data shown were representative of three independent experiments. ^∗^*p* < 0.05; ^∗∗^*p* < 0.01; ^∗∗∗^*p* < 0.001.

We previously reported that increased antioxidant defense is critically required for the acclimation of colorectal cancer cells to acidic extracellular pH [24]. Because autophagy can either reduce or elevate ROS [26–30], we measured the levels of ROS in CRC-AA and their parental cells when autophagy was inhibited. We observed that whereas the ROS levels were increased in CRC-AA cells when exposed to chloroquine (CQ), another autophagy inhibitor, they remained unchanged when treated with ATG5 siRNA (Figures S3a and S3b). These results argue against a direct role of autophagy in reducing ROS in CRC-AA cells. Interestingly, NRF2, the master regulator of cellular antioxidant defense, was even downregulated in CRC-AA cells (Figure 2(a)), suggesting that NRF2 does not contribute to ROS reduction in CRC-AA cells.

NF-*κ*B functions in numerous signaling pathways in response to stress and is broadly involved in cancer progression [31, 32]. It was also reported to be upregulated under acidic condition [33–35]. Importantly, it plays a critical role in maintaining redox homeostasis [36, 37]. We therefore tested whether NF-*κ*B played a role in the adaptation of colorectal cancer cells to acidic environment. We observed that the level of p-p65, the phosphorylated and active form of NF-*κ*B subunit p65, was increased in CRC-AA cells when compared to parental cells (Figure 2(b)). Moreover, p65 was largely localized in nuclei of CRC-AA cells when compared to their parental cells, as shown by Western blot analysis (Figure 2(c)) and immunofluorescence staining (Figure 2(d)). Measurement of mRNA levels of genes encoding representative inflammatory factors by qPCR assay further supported an increased activation of NF-*κ*B in CRC-AA cells compared to parental cells (Figure 2(e)). These data strongly suggest that NF-*κ*B function is enhanced in CRC-AA cells.

### 2.3. CRC-AA Cells Are More Sensitive to NF-*κ*B Inhibition or Depletion

**Figure 3:**
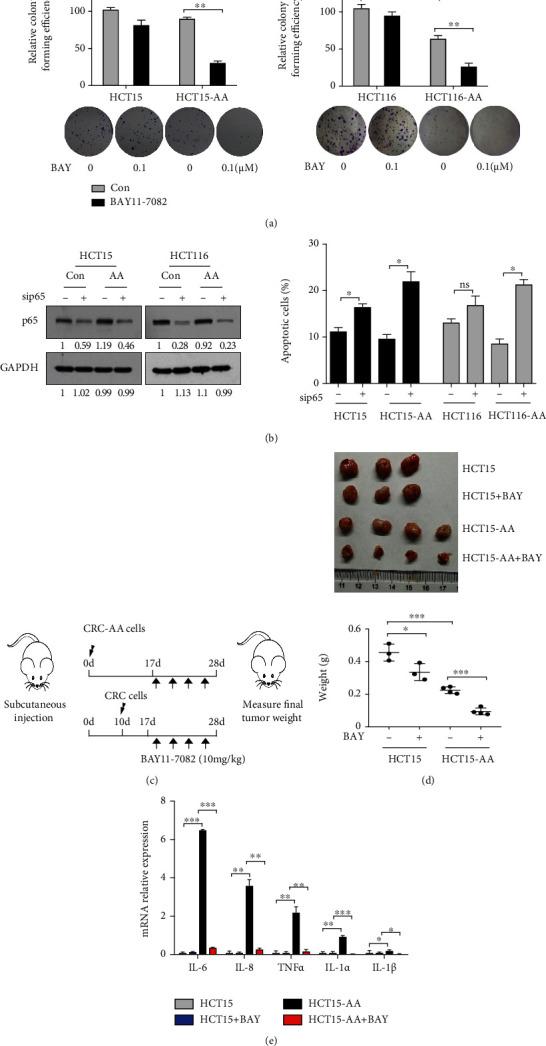
CRC-AA cells are more sensitive to NF-*κ*B inhibition or depletion. (a) Inhibition of colony formation by BAY11-7082 in CRC-AA and their parental cells. Cultured cells were exposed to BAY11-7082 (0.1 *μ*M) for 12 d to allow for colony formation. (b) Depletion of p65 compromised the survival of CRC-AA. Left, knockdown of p65 in HCT15 and HCT116 cells. RNAi efficiency was determined by Western blotting 48 h after transfection with siRNA. GAPDH was used as a loading control. Right, percentages of apoptotic cells under the indicated conditions. (c) Scheme for treatment paradigm of subcutaneous tumor xenografts. Because HCT15-AA cells are less proliferative than their parental cells, they were injected into nude mice 10 days earlier than their parental cells. 17 days later, the tumor-bearing mice were randomized into two groups: vehicle only (DMSO; *n* = 3) and BAY11-7082 only (10 mg/kg; *n* = 4) (given on days 19, 21, 23, and 25 by intraperitoneal injection). Tumor weights were measured at day 28. (d) Tumor xenografts formed by CRC-AA are more sensitive to NF-*κ*B inhibition. (e) mRNA levels of cytokines in tumors for each treatment group, measured by qPCR. ^∗^*p* < 0.05; ^∗∗^*p* < 0.01; ^∗∗∗^*p* < 0.001.

To determine whether the enhanced NF-*κ*B activity is required for the survival and proliferation of CRC-AA, we treated CRC-AA and their parental cells with BAY11-7082, an NF-*κ*B inhibitor, and measured their ability to form colony. While the parental CRC cells were hardly impaired in their colony formation by BAY11-7082 (0.1 *μ*M), the CRC-AA cells exhibited a remarkable reduction in colony formation (Figure 3(a)). BAY11-7082 (5 *μ*M, 48 h) also greatly induced apoptosis in CRC-AA cells while having little effect on their parental cells (Figure S4a). Moreover, depletion of p65 by RNAi led to a more pronounced induction of apoptosis in CRC-AA than in parental CRC cells (Figure 3(b)). These results indicate that NF-*κ*B function is more relied upon for the proliferation and survival of CRC-AA cells than for the parental CRC cells.

To determine whether the CRC-AA cells are also more dependent on NF-*κ*B in vivo, we established a xenograft tumor model in nude mice with HCT15-AA and their parental cells and subjected the mice to BAY11-7082 treatment. Tumor weights were evaluated at the end of the experiment. While the tumors formed by HCT15-AA cells were generally smaller than those by the parental cells, the inhibitory effect of NF-*κ*B inhibitor on the growth of HCT15-AA tumors was more pronounced than on that of CRC tumors (Figure S4b). Considering that smaller tumors might be generally more sensitive to drug treatment than larger tumors, we next compared the sensitivity of HCT15-AA and HCT15 tumors when they reached comparable sizes (Figure 3(c)). We observed that HCT15-AA tumors were again more responsive to NF-*κ*B inhibitor than the HCT15 tumors, with weight reduced by 73% and 42%, respectively (Figure 3(d)). Furthermore, we examined the expression of inflammatory cytokines in the xenograft tumors by qPCR and found that high expression levels of the cytokines in CRC-AA tumors could be drastically reduced by the NF-*κ*B inhibitor (Figure 3(e)).

### 2.4. Enhanced NF-*κ*B Activity Confers Colorectal Cancer Cells Antioxidant Defense

**Figure 4:**
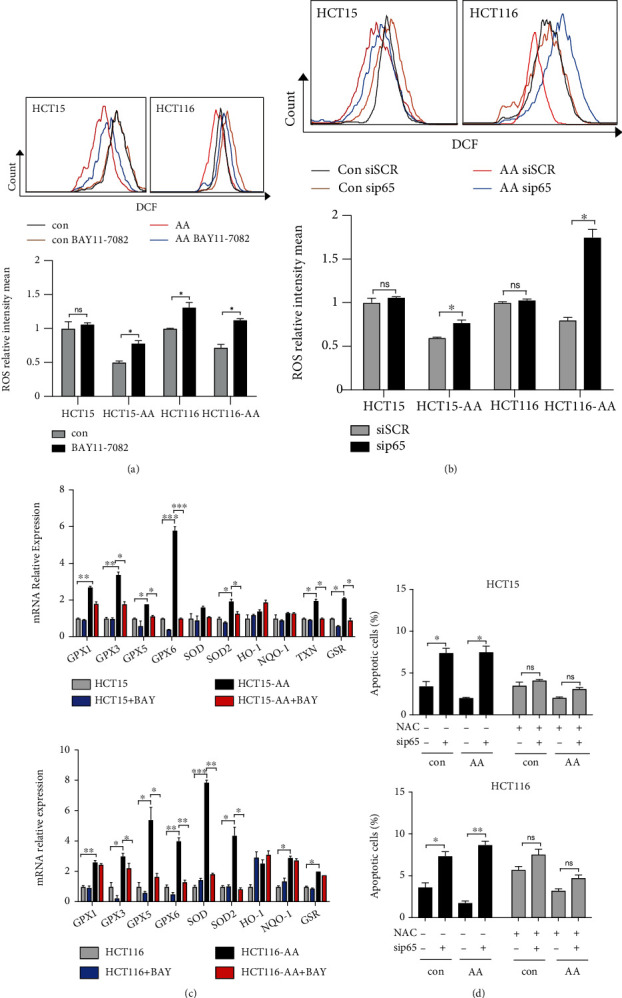
Enhanced NF-*κ*B confers colorectal cancer cells antioxidant defense. (a) Cells were incubated with or without BAY11-7082 (5 *μ*M) for 24 h and then ROS was measured by FACS analysis. (b) ROS was measured by FACS analysis in cells transfected with p65 siRNA for 24 h. (c) mRNA expression of NF-*κ*B-dependent antioxidant genes of CRC and CRC-AA cells treated with BAY11-7082 (5 *μ*M) for 24 h measured by qPCR. (d) NAC attenuates p65 siRNA-induced apoptosis. Cells were transfected with p65 siRNA for 48 h with addition of the NAC (10 mM). ^∗^*p* < 0.05; ^∗∗^*p* < 0.01; ^∗∗∗^*p* < 0.001; ns, *p* > 0.05.

NF-*κ*B is known to activate several antioxidant genes [36, 37]. We therefore tested whether the upregulated NF-*κ*B contributes to the reduction of ROS in CRC-AA cells. We treated CRC-AA and their parental cells with BAY11-7082 for 24 h and then measured the level of ROS by flow cytometry. As shown in Figure 4(a), ROS level was remarkably increased by BAY11-7082 in CRC-AA cells. Similarly, ROS level was significantly increased when p65 was depleted by RNAi (Figure 4(b)). Correspondingly, while the mRNA levels of selected antioxidant target genes downstream of NF-*κ*B were significantly elevated in CRC-AA cells, some of the upregulations were greatly attenuated by BAY11-7082 (Figure 4(c)). Importantly, when CRC-AA cells were pretreated with antioxidant N-acetylcysteine (NAC) for 2 h, the striking induction of apoptosis by p65 RNAi in CRC-AA cells, as shown in Figure 3(b), was completely abolished in the presence of NAC (Figure 4(d) and Figure S4c). These results indicate that CRC-AA cells are more reliant on NF-*κ*B-mediated redox homeostasis for survival under extracellular acidosis than their parental cells.

### 2.5. Upregulation of NF-*κ*B Is Driven by GATA4 in CRC-AA Cells

**Figure 5:**
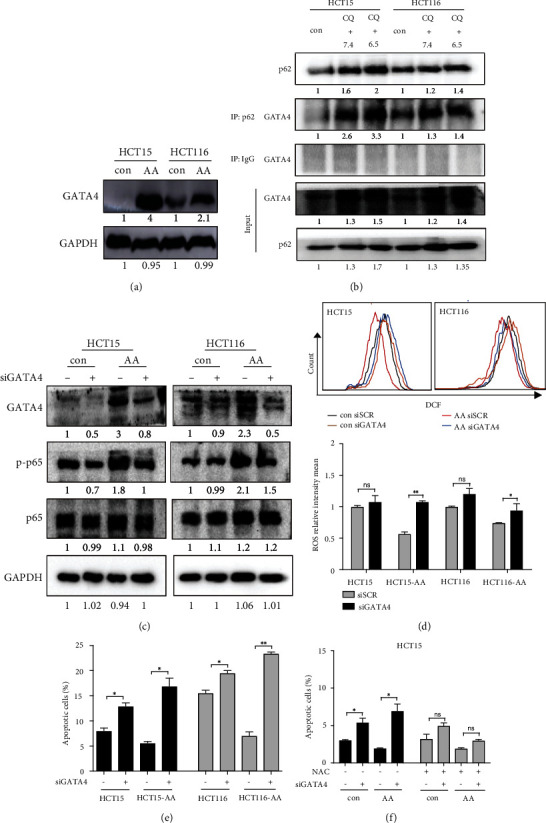
Upregulation of NF-*κ*B is driven by GATA4 in CRC-AA cells. (a) Expression levels of GATA4 determined by Western blotting. (b) Immunoblotting of endogenous GATA4 immunoprecipitated by p62 in CRC cells treated with chloroquine (25 *μ*M) for 24 h under pH 7.4 and pH 6.5 medium, respectively. (c) Knockdown of GATA4 by siRNA for 48 h, p-p65, p65, and GATA4 protein levels were determined by Western blotting. (d) DCF intensity in CRC and CRC-AA cells transfected with GATA4 siRNA for 24 h. (e) Apoptosis was measured by flow cytometry in cells transfected with GATA4 siRNA for 48 h. (f) NAC attenuates GATA4 siRNA-induced apoptosis. Cells were transfected with GATA4 siRNA for 48 h in the presence of the NAC (10 mM). ^∗^*p* < 0.05; ^∗∗^*p* < 0.01; ns, *p* > 0.05.

It was reported that NF-*κ*B is activated by GATA4 during DNA damage-induced cellular senescence and GATA4 itself is degraded by p62-mediated selective autophagy [38]. GATA4 appeared to act, at least in part, through TRAF3IP2 and IL-1A to activate NF-*κ*B and thus sustain senescence-associated secretory phenotype (SASP) [38]. Because there is an enhanced autophagic flux in CRC-AA cells and consequently the diminishment of p62 (Figure 1(b)), we speculated that GATA4 may escape autophagic degradation and thereafter activate NF-*κ*B and augment antioxidant defense in CRC-AA cells. To test this, we determined the level of GATA4 in CRC-AA cells. As we expected, the amount of GATA4 protein was increased in CRC-AA cells (Figure 5(a)), but the GATA4 mRNA level remained unchanged, as determined by qPCR (Figure S5a). To confirm the physical interaction between p62 and GATA4 and to determine whether the interaction might be altered under extracellular acidosis, we immunoprecipitated p62 with the anti-p62 antibody and then measured the amount of GATA4 that binds p62 in CRC cells grown under pH 7.4 and pH 6.5, respectively. We treated CRC cells with chloroquine to block the autophagic flux and enrich p62. A physical interaction between p62 and GATA4 could indeed be detected in CRC cells regardless of the difference in extracellular acidity (Figure 5(b)). Importantly, the level of p-p65 in CRC-AA cells was reduced when GATA4 was depleted by RNAi (Figure 5(c)). Moreover, when CRC-AA and their parental cells were depleted of GATA4 by siRNA, the expression of inflammatory cytokines was significantly decreased (Figure S5b). Consistently, the amount of p65 localized in the nucleus was reduced by GATA4 depletion in CRC-AA cells (Figure S5c). Together, these data indicate that like its role in promoting SASP in DNA damage-induced cellular senescence, the GATA4 elevation is also responsible for the upregulation of NF-*κ*B in CRC-AA cells.

If the GATA4-NF-*κ*B axis is required for the survival and antioxidant defense in CRC-AA cells, it is expected that depletion of GATA4 would similarly compromise their survival and antioxidant defense. Indeed, the levels of ROS (Figure 5(d)) and apoptosis (Figure 5(e)) were significantly increased in CRC-AA when GATA4 was depleted. Importantly, the increased apoptosis caused by GATA4 depletion in CRC-AA cells could be blocked by NAC (Figure 5(f) and Figure S5d). Taken together, these data indicate that GATA4 upregulates NF-*κ*B in CRC-AA cells and this axis promotes the survival of CRC cells in acidic microenvironment by conferring increased antioxidant defense.

**Figure 6:**
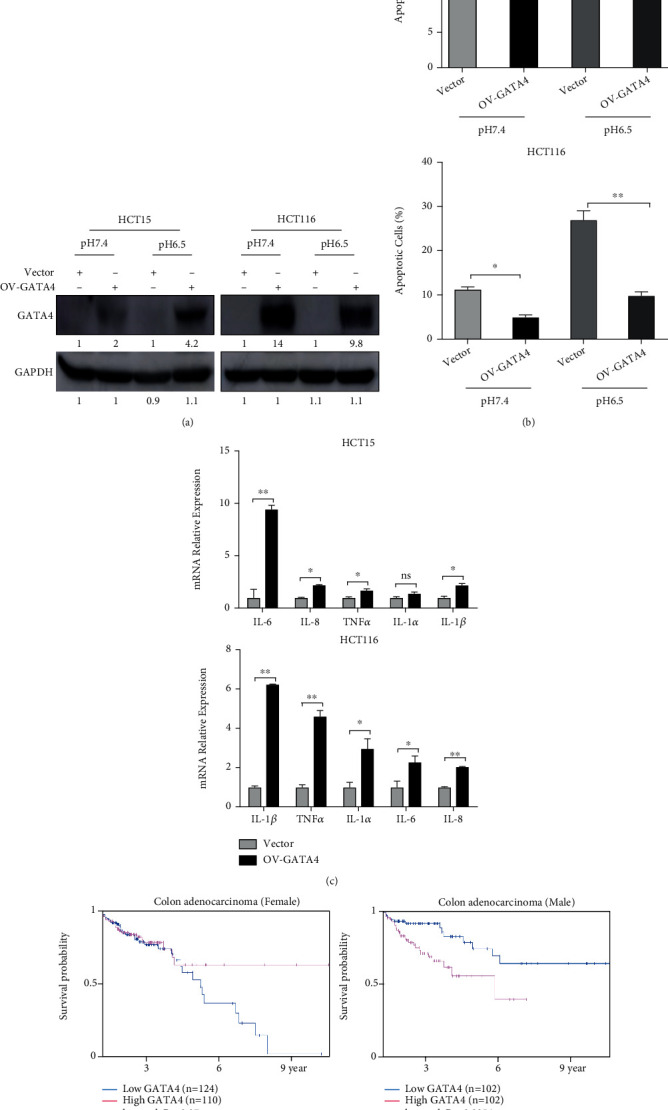
Overexpression of GATA4 in HCT15 cells. (a) HCT15 cells were transfected with Teto-GATA4 or vector and cultured with pH 7.4 or pH 6.5 medium for 72 h. GATA4 protein level was determined by Western blotting. (b) Apoptosis levels measured by flow cytometry. (c) mRNA expression of inflammatory cytokines measured by qPCR. (d) Kaplan-Meier analysis of survival probability in colon adenocarcinoma patients. ^∗^*p* < 0.05; ^∗∗^*p* < 0.01; ns, *p* > 0.05.

If the robust autophagic flux in CRC-AA cells drives the upregulation of the GATA4-NF-*κ*B axis and consequently supports the increased survival, via the reduction of p62, it is expected that depletion of p62 would render CRC cells resistant to acidic microenvironment. Indeed, when p62 was knocked down by RNAi, the levels of GATA4 and p-p65 were greatly increased in CRC cells (Figure S6a). Importantly, CRC cells treated with p62 siRNA exhibited a lower apoptotic rate under pH 6.5 than control cells (Figure S6b). These results indicate that the prosurvival GATA4-NF-*κ*B axis unleashed by p62 depletion contributes to the adaptation of CRC cells to extracellular acidosis. Furthermore, we tested the function of GATA4 by overexpressing GATA4 in CRC cells (Figure 6(a)). CRC cells overexpressing GATA4 survived better under pH 6.5 than control cells (Figure 6(b)). They also expressed higher levels of inflammatory cytokines (Figure 6(c)). To assess the clinical significance of GATA4 in colon cancer, we analyzed the relationship between GATA4 expression and survival probability. An analysis of human colon cancer samples data from The Cancer Genome Atlas (TCGA) showed that higher GATA4 expression correlated with shorter survival in male patients with colon adenocarcinoma (*p* = 0.0054), though no such correlation was detected in females (*p* = 0.27; Figure 6(d)).

### 2.6. ICAM-1 Secreted by Cancer Cells Promotes Survival under Acidic Microenvironment

**Figure 7:**
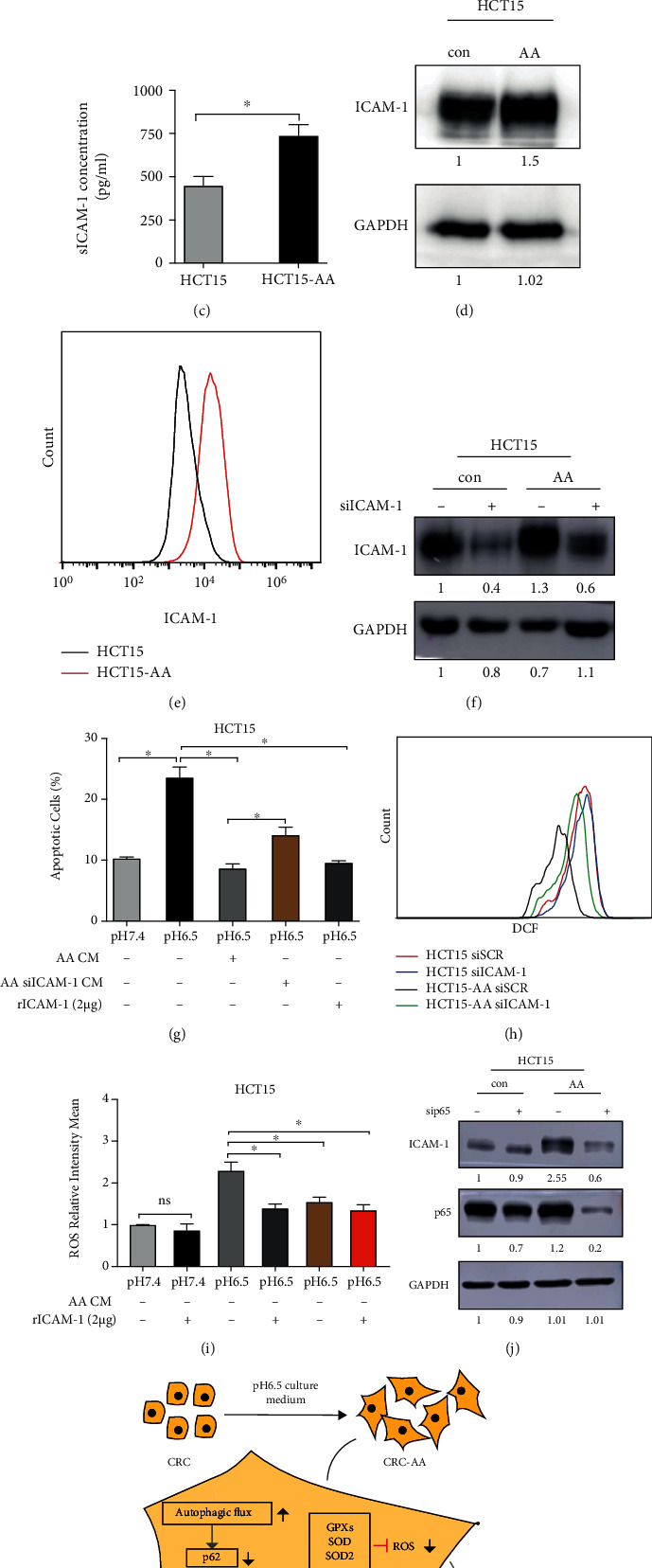
ICAM-1 secreted by colon cancer cells promotes survival under acidic microenvironment. (a) First row, HCT15 cells were cultured in pH 7.4 or pH 6.5 medium; second row, HCT15 cells were cultured in pH 6.5 medium with 1/3 pH 7.4 or pH 6.5 condition medium and measured with colony-forming assay after 12 d. (b) HCT15 cells were treated as in (a), and apoptosis was measured by flow cytometry. (c) Soluble ICAM-1 concentration was measured by ELISA. (d) Expression of ICAM-1 was determined by Western blotting. (e) Flow cytometric analysis of ICAM-1 levels in CRC-AA and their parental cells. (f) Knockdown of ICAM-1. HCT15 and HCT15-AA cells were transfected with siRNA for 48 h; RNAi efficiency was determined by Western blotting. GAPDH was used as a loading control. (g) HCT15 cells were cultured in pH 7.4 or pH 6.5 medium, with addition of CRC-AA conditioned medium, CRC-AA siICAM-1 conditioned medium, or ICAM-1 recombinant protein. The level of apoptosis was measured by flow cytometry. (h) DCF intensity in CRC and CRC-AA cells transfected with ICAM-1 siRNA for 24 h. (i) HCT15 cells were cultured in pH 7.4 or pH 6.5 medium, with addition of CRC-AA conditioned medium or ICAM-1 recombinant protein; ROS levels were measured by flow cytometry analysis of DCF. (j) NF-*κ*B-dependent expression of ICAM-1. p65 was depleted by siRNA for 48 h in HCT15 and HCT15-AA cells. ICAM-1 and p65 protein levels were determined by Western blotting. (k) Schematic model showing the role of the GATA4-NF-*κ*B pathway driven by autophagy in CRC-AA cells. ^∗^*p* < 0.05; ^∗∗^*p* < 0.01; ns, *p* > 0.05.

Because NF-*κ*B transactivates genes that encode a large number of soluble factors, we wondered whether NF-*κ*B, besides their antioxidant function, may also promote cancer cell survival in an autocrine or paracrine fashion. To explore this, we determined the colony-forming ability of CRC cells in pH 6.5, with or without the addition of medium conditioned by CRC-AA cells. The results showed that colony formation by CRC cells was greatly increased when HCT15-AA conditioned medium was added (Figure 7(a)). However, the medium conditioned by HCT116-AA was less effective (Figure S7a). HCT15-AA conditioned medium could also reduce the level of apoptosis in HCT15 cells under pH 6.5 (Figure 7(b)). These results indicated that factors secreted by HCT15-AA cells have protective effects on cells exposed to acidic environment. To screen for the factors that are responsible for the protective effect of the conditioned medium, we subjected HCT15-AA and their parental cells to protein chip analysis. We found that ICAM-1 was significantly elevated in HCT15-AA cells than in HCT15 cells (Supplementary Table 2). We further confirmed the upregulation of ICAM-1 by ELISA (Figure 7(c)), Western blot (Figure 7(d)), and flow cytometry (Figure 7(e)) in HCT15-AA cells when compared to their parental cells. Importantly, the protective effect conferred by the conditioned medium of HCT15-AA cells was significantly attenuated when ICAM-1 was depleted by RNAi (Figures 7(f) and 7(g)). Consistent with the lesser protective effect of HCT116-AA conditioned medium, the ICAM-1 expression level was much lower in HCT116-AA cells than in HCT15-AA cells (Figure S7b). Addition of recombinant ICAM-1 could also protect the HCT15 cells from apoptosis in the acidic growth medium (Figure 7(g)). Interestingly, depletion of ICAM-1 increased the ROS level more drastically in HCT15-AA cells than in their parental cells (Figure 7(h)). Both the HCT15-AA conditioned medium and recombinant ICAM-1 could reduce the ROS levels in HCT15 cells under low pH 6.5 (Figure 7(i)). To determine whether the upregulation of ICAM-1 in HCT15-AA cells was mediated by NF-*κ*B, we depleted p65 by RNAi and found that the protein level of ICAM-1 was significantly reduced by p65 depletion (Figure 7(j)). Thus, the GATA4-NF-*κ*B axis can also promote the survival of CRC-AA cells under acidic microenvironment by augmenting the production of prosurvival autocrine factors.

## 3. Discussion

Cancer cells cannot only survive under acidic microenvironment but also become more malignant. How cancer cells become adapted to or are selected for extracellular acidosis remains to be fully elucidated. Consistent with reports that chronic autophagy is an adaptive response of cancer cells to acidic microenvironment [20, 21], the acidosis-acclimated colorectal cancer cells also exhibit an increased autophagic flux. We previously reported that colorectal cancer cells that have become adapted to acidic microenvironment exhibit reduced level of ROS when compared to their parental cells [24]. Because autophagy plays a critical role in the clearance of damaged organelles, including endoplasmic reticulum and mitochondria and oxidized macromolecules [39–41], the increased autophagy in acidosis-acclimated cancer cells is expected to contribute to the reduction of ROS in CRC-AA cells. However, NRF2, which transactivates large number of genes responsible for antioxidant defense and detoxification, was even greatly reduced in CRC-AA cells. We believe that the downregulation of NRF2 in CRC-AA cells may have been caused by the low maintenance of p62 level, due to the increased autophagic flux. As a substrate adaptor of CUL3-based ubiquitin E3 ligase complex, KEAP1 promotes the ubiquitination and proteasomal degradation of NRF2 [42, 43]. The p62/SQSTM1 can compete with NRF2 for KEAP1 binding and thus spare NRF2 from degradation [28, 44]. With the decline of p62 in CRC-AA cells, NRF2 becomes more easily targeted by KEAP1. Thus, NRF2 is unlikely to contribute to the antioxidant defense in acidosis-acclimated cancer cells.

NF-*κ*B serves as a master regulator of cellular response to stress [31, 32]. It can transactivate a large number of antioxidant and prooxidant genes to regulate the amount of ROS [36, 37]. We found that NF-*κ*B is upregulated in CRC-AA cells and is more relied upon for their survival. This finding is consistent with previous reports [31–35]. While inhibition of NF-*κ*B or depletion of p65 resulted in a significant increase in cell death in CRC-AA cells, the deleterious effect of NF-*κ*B functional impairment on CRC-AA cells was abrogated in the presence of antioxidant NAC. The upregulation of NF-*κ*B further argues against the role of NRF2 in antioxidant defense in CRC-AA cells because the NRF2-ARE pathway is antagonized by NF-*κ*B [45]. It was recently reported that increased autophagy in response to DNA damage led to the decline of p62 that is required for the autophagic degradation of GATA4 [38]. The persistence of high level of GATA4 due to the declined p62 can activate NF-*κ*B and drive SASP in DNA damage-induced cellular senescence. Cellular senescence, which confers cells resistance to the induction of apoptosis, can be regarded as a prosurvival strategy. We demonstrate here that an increased autophagic flux in CRC-AA cells also unleashes the GATA4-NF-*κ*B pathway to support cell survival under the acidic microenvironment. CRC-AA cells generally have a lower rate of apoptosis and are highly sensitive to the blockade of autophagy. High level of ROS is observed in cancer cells acutely exposed to acidic medium and is associated with increased cell death, which can be offset by increased autophagic flux, as shown in this study and reported previously [24, 46]. While this study demonstrated a critical role of NF-*κ*B in cell survival via its antioxidant function, the classical NF-*κ*B targets such as COX2 and iNOS are also known to promote the survival of colon cancer cells [47, 48]; therefore, further studies are needed to determine the other NF-*κ*B targets that also contribute to cell survival under acidosis.

Depletion of GATA4 severely affected the survival of CRC-AA cells in a ROS-dependent manner. Interestingly, GATA4 is amplified in esophageal adenoma and gastric cancer and possesses oncogenic properties [49, 50]. Through Kaplan-Meier analysis of colon adenocarcinoma in TCGA database, it was revealed that a higher GATA4 expression is associated poorer prognosis in CRC males, but not in females. It is worth noting that both HCT15 and HCT116 cell lines were derived from males. In fact, the ratio of incidence rate of colon cancer in male and female population is about 2-3 : 1. Why GATA4 expression level is associated with prognosis only in males needs further study.

NF-*κ*B promotes the production of numerous cytokines and multiple adhesion molecules [51]. We observed that HCT15-AA cells can produce soluble ICAM-1 to promote their own survival under acidic condition in an autocrine manner. Interestingly, ICAM-1 also possesses antioxidant effect. However, consistent with the very low expression of ICAM-1 in HCT116 and HCT116-AA cells, the HCT116-AA supernatant was less effective in promoting cancer cell survival under acidic pH, suggesting that autocrine effect of CRC-AA cells may not apply to all cancer cells. Nevertheless, our findings demonstrated that the upregulation of GATA4-NF-*κ*B pathway, as a consequence of increased autophagic flux, plays a critical role for cancer cell survival under acidic environments (Figure 7(k)). The heavy reliance of cancer cells on autophagy, NF-*κ*B, and antioxidant defense under acidic microenvironment indicates that each of those chain links can be targeted for cancer therapy.

Accumulation of p62, which functions as a receptor as well as a substrate of autophagy, appears to act as signaling hub for many cellular processes and have distinct consequences depending on the contexts [52]. It can induce hepatocellular carcinoma by promoting the activation of NRF2 and mTORC1 and the upregulation of c-Myc [53]. However, p62 can also have a deleterious effect on cell survival. For example, p62 was reported to directly inhibit RNF168 E3 ligase activity that is required for histone ubiquitination and the subsequent recruitment of DNA repair factors, and cells with defective autophagy, consequently abnormal accumulation of p62, are more sensitive to ionizing radiation [54]. Depletion of p62 could rescue impaired tumor growth. We showed here that the p62 diminishment due to increased autophagic flux enabled the CRC-AA cells to gain a survival advantage that is endowed by the GATA4-NF-*κ*B axis. A similar mechanism may operate in other contexts where autophagy serves as prosurvival strategy. A recent study shows that colorectal cancer cells enter a diapause-like drug-tolerant persister (DTP) state in response to chemotherapy [55]. Those cancer cells in DTP state are also dependent on autophagy for survival. It would be interesting to determine whether the DTP state is also associated with an upregulation of the GATA4-NF-*κ*B axis.

In conclusion, our findings support a model wherein under chronic acidic microenvironment, colorectal cancer cells are particularly reliant on the antioxidant p62-GATA4-NF-*κ*B axis for survival. Targeting of this prosurvival axis should be further explored as a cancer therapeutic strategy.

## 4. Materials and Methods

### 4.1. Cell Culture

Human colorectal cancer (CRC) cell lines HCT15 and HCT116 were obtained from the Cell Bank of Chinese Academy of Sciences (Shanghai, China). The cells were cultured in RPMI-1640 (pH 7.4), supplemented with 10% fetal bovine serum (FBS), 100 U/mL penicillin, and 100 *μ*g/mL streptomycin. Cells were maintained in a 5% CO_2_/95% air incubator at 37°C. Acidic medium was prepared by adjusting the pH to 6.5 with 25 mmol/L each of PIPES and HEPES. CRC-AA cells (HCT15-AA and HCT116-AA) were obtained by continuously culturing and passing the CRC cells in acidic medium for at least three months.

### 4.2. Transmission Electron Microscopy

Cells were harvested and fixed for 24 h at 4°C in 3% pentanediol. Cells were washed with phosphate buffer and subsequently postfixed in 1% (*v*/*w*) osmium tetroxide (Merck). Samples were dehydrated by successive passages in increasing concentrated ethanol baths (30, 50, 70, 85, and 100%). After embedding in epon resin LX 112 (Ladd Research Industries), ultrathin sections of cell-covered filters were prepared using an 8800 ultrotome III (LKB). TEM analysis used TEM grids (Agar Scientific) covered with nonporous formvar.

### 4.3. Antibodies

Cells were harvested and lysed in WIP lysis buffer (Beyotime, Shanghai, China) for immunoblots and immunoprecipitation. Protein concentrations of the lysates were determined by the BCA protein assay system (Beyotime). Equal amounts of protein were separated by 10% SDS-PAGE, transferred to PVDF membrane (Millipore, Billerica, MA). The used antibodies were anti-LC3B (No. 3868, Cell Signaling Technology, 1 : 1000); anti-ATG5 (10181-2-AP, Proteintech, 1 : 1000); anti-ATG7 (ab133528, Abcam, 1 : 50,000); anti-p62 (ab56416, Abcam, 1 : 2000); anti-p62 (ab155686, Abcam, 1 : 2000); anti-BECN1 (No. 3495, Cell Signaling Technology, 1 : 1000); anti-NRF2 (ab137550, Abcam, 1 : 1000); anti-GATA4 (ab12465, Abcam, 1 : 1000); anti-p65 (No. 8242, Cell Signaling Technology, 1 : 2000); anti-p-p65 (No. 3033, Cell Signaling Technology, 1 : 2000); anti-GAPDH (sc-365062, Santa Cruz, 1 : 1000); anti-ICAM-1 (sc-8439, Santa Cruz, 1 : 1000); anti-eIF2*α* (No. 5324, Cell Signaling Technology, 1 : 1000); anti-p-eIF2*α* (No. 3398, Cell Signaling Technology, 1 : 1000); anti-Chop (No. 5554, Cell Signaling Technology, 1 : 1000); and anti-ATF4 (10835-1-AP, Proteintech, 1 : 1000). Protein A/G was purchased from Thermo Fisher, rab-IgG from Beyotime, horseradish peroxidase secondary antibody from Amersham Pharmacia Biotech, and ECL kit from Thermo Fisher.

### 4.4. Quantitative RT-PCR

Total RNA was collected by using TRIzol reagent (Invitrogen, China) according to the manufacturer's protocol. cDNA was synthesized by reverse transcription of 1 *μ*g of total RNA with random hexamers. Real-time quantitative PCR was performed using the LightCycler® 480 sequence Detection System (Roche Applied Science, Germany) with SYBR-Green (Invitrogen). The primer sequences are listed in Supplementary Table 1. Each assay was normalized to the level of GAPDH mRNA.

### 4.5. RNA Interference

Cells were transfected with siRNAs (50 nM) using Lipofectamine 2000 (Invitrogen, USA) according to the manufacturer's instruction. ATG5: 5′-CAAUUGGUUUGCUAUUUGA-3′; p65: 5′-GAUGAGAUCUUCCUACUGU-3′; GATA4: 5′-CGAAUGACGGCAUCUGUUU-3′; ICAM-1: 5′-GACAUAUGCCAUGCAGCUA-3′; p62: 5′-AGAUUCGCCGCUUCAGCUUTT-3′. RNAi efficiency was determined 48 h after transfection. GATA4 plasmid was purchased from Addgene (#49535).

### 4.6. Flow Cytometry Analysis of Apoptosis

Apoptotic cells were determined using the Annexin V/Dead Cell Apoptosis Kit (Invitrogen). Cells were harvested using 0.25% Trypsin-EDTA, centrifuged (300 *g*), and washed twice in PBS. Cells were resuspended in 100 *μ*L of 1x binding buffer at a density of 1 × 10^6^ cells/mL and incubated in the dark with annexin-V-fluorescein isothiocyanate (or APC) and propidium iodide (or 7-AAD). Cell fluorescence was assessed in a FACScan flow cytometer (Becton Dickinson, San Jose, CA, USA).

### 4.7. Immunofluorescence

Cells grown on cover slips in 6-well plate were washed in PBS twice and were fixed in 4% paraformaldehyde for 15 min at room temperature. Cells were then treated with 0.2% Triton X-100 in PBS for 15 min and then blocked with 10% normal goat serum in PBS for 60 min, following which rabbit anti-p65 antibody (Cell Signaling Technology) was added at a dilution of 1 : 400 in 5% normal goat serum in PBS and incubated overnight at 4°C. The cover slips were washed and incubated for one hour in the dark with the Rhodamine-labeled secondary antibody at a dilution of 1 : 200 in 5% normal goat serum in PBS. Cells were washed four times in PBS. The nuclei were counterstained in DAPI and were mounted with nail polish. Slides were then examined under a fluorescence microscope.

### 4.8. Clonogenic Survival Assay

Cells were trypsinized and suspended in complete medium, counted, and replated in 100 mm tissue culture dishes to allow formation of macroscopic colonies. Plates were incubated in a 5% CO_2_/95% air incubator at 37°C for 10 to 14 days, fixed with methanol, and stained with Giemsa, and colonies containing at least 50 cells in size were counted.

### 4.9. Tumor Xenograft

Four-week-old male nude mice were purchased from Nanjing Experimental Animal Center and kept in pathogen-free conditions and handled in accordance with the requirements of the Guideline for Animal Experiments. Mice were inoculated with CRC and CRC-AA cells (8 × 10^6^ cells suspended in 100 *μ*L PBS for each mouse) at the same date or at different dates (CRC-AA inoculated earlier) so that the tumors formed by CRC-AA and CRC cells can reach the similar sizes at the time of BAY11-7082 treatment. In the latter scheme, CRC cells were inoculated 10 days later than the CRC-AA cells. Mice bearing CRC or CRC-AA were each randomized into two treatment groups: vehicle only (DMSO) and BAY11-7082 only (10 mg/kg) (given on days 19, 21, 23, and 25 by intraperitoneal injection). Tumor weights were measured at day 28.

### 4.10. Determination of Cellular ROS

ROS was measured using Reactive Oxygen Species Assay Kit (Beyotime Biotechnology, Shanghai, China) according to the manufacturer's protocols. In brief, cells were harvested and washed with PBS and then labeled with 10 *μ*M DCFH-DA probe for 15 min at 37°C. The labeled cells were washed with PBS. ROS was examined by flow cytometry (FACSCanto II, BD Biosciences), and 10,000 viable cells were analyzed in each measurement.

### 4.11. Analysis of TCGA Data

The result in Figure 6(d) is based on data generated by TCGA Research Network: https://www.proteinatlas.org/. The *p* value for Kaplan-Meier plot shows results from analysis of correlation between mRNA expression level and patient survival.

### 4.12. Protein Array Analysis

Cells were expanded in a 5% CO_2_/95% air incubator at 37°C and harvested. The protein signal intensity was quantified and normalized with positive protein controls. Differentially expressed proteins were identified by their standardized values. The protein array assay was performed by Aksomics (Shanghai, China).

### 4.13. Measurement of ICAM-1 Expression

ICAM-1 in the supernatant of cultured cells was measured using ICAM-1 ELISA kit (ab174445, Abcam). ICAM-1 on cell surface was determined by flow cytometry. Cells were stained with ICAM-1 antibodies (Santa Cruz, 1 : 1000) and incubated for 15 min. The stained ells were washed twice with and resuspended in PBS and subjected to flow cytometry analysis on BD FACSCanto II, and data were analyzed with FlowJo (TreeStar, USA).

### 4.14. Statistical Analysis

Student's *t*-test was used to determine the statistical significance between experimental groups. Difference was considered significant if the *p* value was less than 0.05.

## Data Availability

Additional data used to support the findings of this study are available from the corresponding author upon request.

## Supplementary Materials

Supplementary Materials Figure S1: Autophagy flux was blocked by lysosomal inhibitor in CRC and CRC-AA cells. Figure S2: ER stress marker expressions in CRC and CRC-AA cells. Figure S3: The role of autophagy in reducing ROS in CRC-AA cells. Figure S4: CRC-AA cells are more sensitive to NF-*κ*B inhibition or depletion. Figure S5: Upregulation of NF-*κ*B is driven by GATA4 in CRC-AA cells. Figure S6: p62 depletion promotes CRC cell survival under acidic microenvironment. Figure S7: ICAM-1 expression in HCT116 cells. Table S1: Primers for RT quantitative PCR. Table S2: Protein array results.
