# Supplementary material for: Autophagic Flux Unleashes GATA4-NF-κB Axis to Promote Antioxidant Defense-Dependent Survival of Colorectal Cancer Cells under Chronic Acidosis
Source: Oxid Med Cell Longev. 2021 Dec 26;2021:8189485. doi: 10.1155/2021/8189485 (PMC8720590; doi:10.1155/2021/8189485)
Supplement: Supplementary Materials — Figure S1: Autophagy flux was blocked by lysosomal inhibitor in CRC and CRC-AA cells. Figure S2: ER stress marker expressions in CRC and CRC-AA cells. Figure S3: The role of autophagy in reducing ROS in CRC-AA cells. Figure S4: CRC-AA cells are more sensitive to NF-κB inhibition or depletion. Figure S5: Upregulation of NF-κB is driven by GATA4 in CRC-AA cells. Figure S6: p62 depletion promotes CRC cell survival under acidic microenvironment. Figure S7: ICAM-1 expression in HCT116 cells. Table S1: Primers for RT quantitative PCR. Table S2: Protein array results. [file 8189485.f1.zip › Table S2.pdf]

Table S2. Protein Array Results

Name	specific value (HCT15-AA vs HCT15)	p value (HCT15-AA vs HCT15)
ICAM-1	4.996840817	0.001938699
ICAM-3	2.232779739	0.014843475
MSP-α	1.781015941	0.020487589
BLC	1.803473899	0.052207912
TIMP-2	5.364782749	0.060255229
IGFBP-6	1.462668632	0.060209282
MIP-1α	1.366631288	0.075853214
bFGF	1.640803264	0.080914442
BTC	3.094803984	0.110255547
TIMP-1	1.953230353	0.121005803
uPAR	1.933061007	0.374371624
