# Supplementary material for: Autophagic Flux Unleashes GATA4-NF-κB Axis to Promote Antioxidant Defense-Dependent Survival of Colorectal Cancer Cells under Chronic Acidosis
Source: Oxid Med Cell Longev. 2021 Dec 26;2021:8189485. doi: 10.1155/2021/8189485 (PMC8720590; doi:10.1155/2021/8189485)
Supplement: Supplementary Materials — Figure S1: Autophagy flux was blocked by lysosomal inhibitor in CRC and CRC-AA cells. Figure S2: ER stress marker expressions in CRC and CRC-AA cells. Figure S3: The role of autophagy in reducing ROS in CRC-AA cells. Figure S4: CRC-AA cells are more sensitive to NF-κB inhibition or depletion. Figure S5: Upregulation of NF-κB is driven by GATA4 in CRC-AA cells. Figure S6: p62 depletion promotes CRC cell survival under acidic microenvironment. Figure S7: ICAM-1 expression in HCT116 cells. Table S1: Primers for RT quantitative PCR. Table S2: Protein array results. [file 8189485.f1.zip › TableS1.pdf]

Table S1. Primers for RT quantitative PCR

Gene ID	Primers	Sequences（5'→3'）	Gene ID	Primers	Sequences（5'→3'）
3569	IL-6-RT forward<br>IL-6-RT reverse	ACTCACCTCTTCAGAACGAATTG<br>CCATCTTTGGAAGGTTCAGGTTG	2936	GSR-RT forward<br>GSR-RT reverse	CACTTGCGTGAATGTTGGATG<br>TGGGATCACTCGTGAAGGCT
2597	GAPDH-RT forward<br>GAPDH-RT reverse	GGAGCGAGATCCCTCCAAAAT<br>GGCTGTTGTCATACTTCTCATGG	6648	SOD2-RT forward<br>SOD2-RT reverse	GCTCCGGTTTTGGGGTATCTG<br>GCGTTGATGTGAGGTTCCAG
3586	IL-10-RT forward<br>IL-10-RT reverse	GACTTTAAGGGTTACCTGGGTTG<br>TCACATGCGCCTTGATGTCTG	8678	BECN1-real time F<br>BECN1-real time R	CCATGCAGGTGAGCTTCGT<br>GAATCTGCGAGAGACACCATC
3576	IL-8-RT forward<br>IL-8-RT reverse	TTTTGCCAAGGAGTGCTAAAGA<br>AACCCTCTGCACCCAGTTTTC	9474	ATG5-real time F<br>ATG5-real time R	AAAGATGTGCTTCGAGATGTGT<br>CACTTTGTCAGTTACCAACGTCA
7124	TNF-α-RT forward<br>TNF-α-RT reverse	CCTCTCTCTAATCAGCCCTCTG<br>GAGGACCTGGGAGTAGATGAG	81631	LC3B-real time F<br>LC3B-real time R	GATGTCCGACTTATTCGAGAGC<br>TTGAGCTGTAAGCGCCTTCTA
3552	IL-1α-RT forward<br>IL-1α-RT reverse	TGGTAGTAGCAACCAACGGGA<br>ACTTTGATTGAGGGCGTCATTC	9140	ATG12-real time F<br>ATG12-real time R	CTGCTGGCGACACCAAGAAA<br>CGTGTTCGCTCTACTGCCC
3553	IL-1β-RT forward<br>IL-1β-RT reverse	ATGATGGCTTATTACAGTGGCA<br>GTCGGAGATTCGTAGCTGGA	5293	PIK3CD-real time F<br>PIK3CD-real time R	AAGGAGGAGAATCAGAGCGTT<br>GAAGAGCGGCTCATACTGGG
4843	iNOS-RT forward<br>iNOS -RT reverse	CAGGAGGAGAGAGATCCGATTTA<br>GCATTAGCATGGAAGCAAAGA	7295	TXN-real time F<br>TXN-real time R	GTGAAGCAGATCGAGAGCAAG<br>CGTGGCTGAGAAGTCAACTACTA
6647	SOD-RT forward<br>SOD-RT reverse	GGTGGGCCAAAGGATGAAGAG<br>CCACAAGCCAAACGACTTCC	3162	HO-1-real time F<br>HO-1-real time R	AAGACTGCGTTCCTGCTCAAC<br>AAAGCCCTACAGCAACTGTCG
2876	GPX1-RT forward<br>GPX1-RT reverse	CAGTCGGTGTATGCCTTCTCG<br>GAGGGACGCCACATTCTCG	1728	NQO-1-real time F<br>NQO-1-real time R	GAAGAGCACTGATCGTACTGGC<br>GGATACTGAAAGTTCGCAGGG
2878	GPX3-RT forward<br>GPX3-RT reverse	AGAGCCGGGGACAAGAGAA<br>ATTTGCCAGCATACTGCTTGA	2626	GATA4-real time F<br>GATA4-real time R	CGACACCCCAATCTCGATATG<br>GTTGCACAGATAGTGACCCGT
2880	GPX5-RT forward<br>GPX5-RT reverse	ATGACTACACAGTTAAGGGTCGT<br>GGATATTGCGCTGTCAGACCA			
257202	GPX6-RT forward<br>GPX6-RT reverse	CAAAGGGGTAACAGGCACCAT<br>GGCGGCCACATTGACAAAC			
