# Supplementary figures and images for: Autophagic Flux Unleashes GATA4-NF-κB Axis to Promote Antioxidant Defense-Dependent Survival of Colorectal Cancer Cells under Chronic Acidosis
Source: Oxid Med Cell Longev. 2021 Dec 26;2021:8189485. doi: 10.1155/2021/8189485 (PMC8720590; doi:10.1155/2021/8189485)

# Supplementary Figure 1

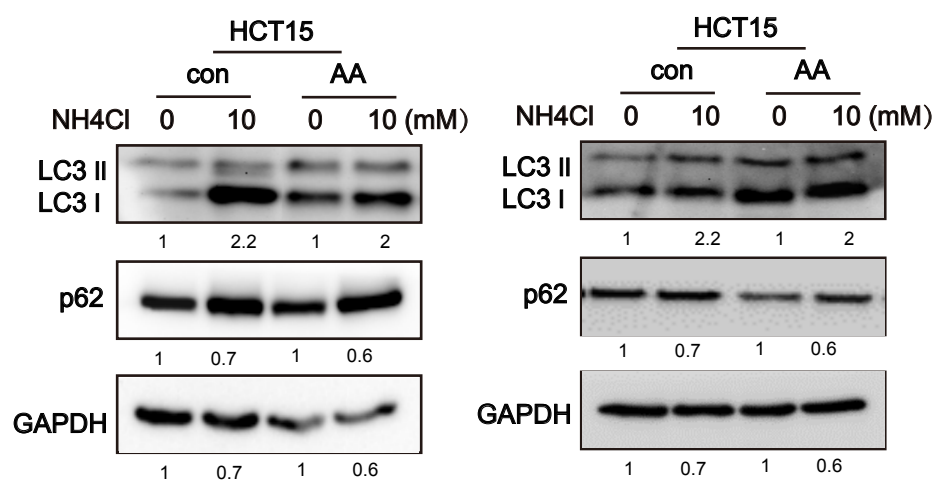

(a)

Supplement: Supplementary Materials — Figure S1: Autophagy flux was blocked by lysosomal inhibitor in CRC and CRC-AA cells. Figure S2: ER stress marker expressions in CRC and CRC-AA cells. Figure S3: The role of autophagy in reducing ROS in CRC-AA cells. Figure S4: CRC-AA cells are more sensitive to NF-κB inhibition or depletion. Figure S5: Upregulation of NF-κB is driven by GATA4 in CRC-AA cells. Figure S6: p62 depletion promotes CRC cell survival under acidic microenvironment. Figure S7: ICAM-1 expression in HCT116 cells. Table S1: Primers for RT quantitative PCR. Table S2: Protein array results. [file 8189485.f1.zip › sup figure 1.pdf]

## Supplementary Figure 2

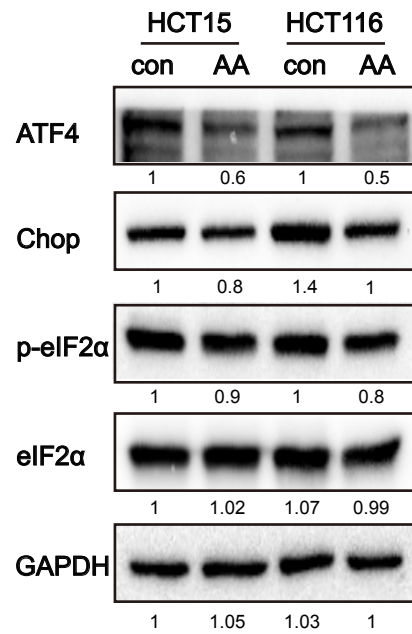

(a)

Supplement: Supplementary Materials — Figure S1: Autophagy flux was blocked by lysosomal inhibitor in CRC and CRC-AA cells. Figure S2: ER stress marker expressions in CRC and CRC-AA cells. Figure S3: The role of autophagy in reducing ROS in CRC-AA cells. Figure S4: CRC-AA cells are more sensitive to NF-κB inhibition or depletion. Figure S5: Upregulation of NF-κB is driven by GATA4 in CRC-AA cells. Figure S6: p62 depletion promotes CRC cell survival under acidic microenvironment. Figure S7: ICAM-1 expression in HCT116 cells. Table S1: Primers for RT quantitative PCR. Table S2: Protein array results. [file 8189485.f1.zip › sup figure 2.pdf]

# Supplementary Figure 3

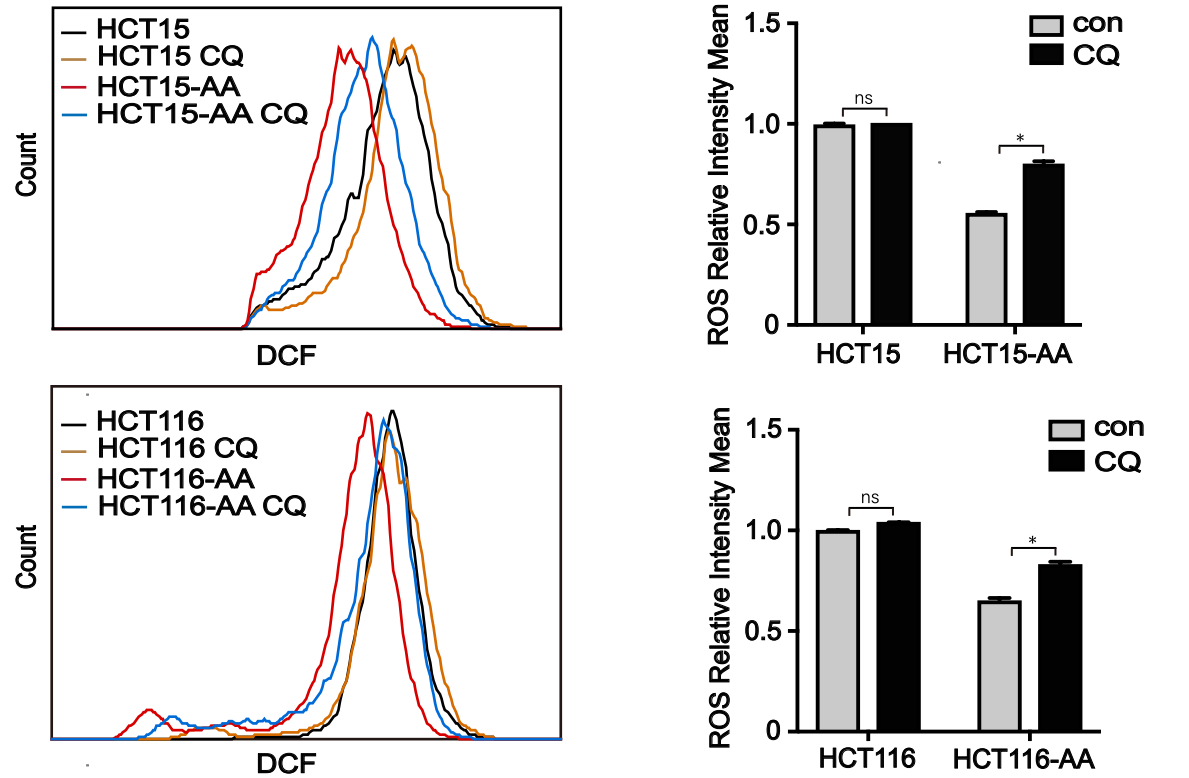

(a)

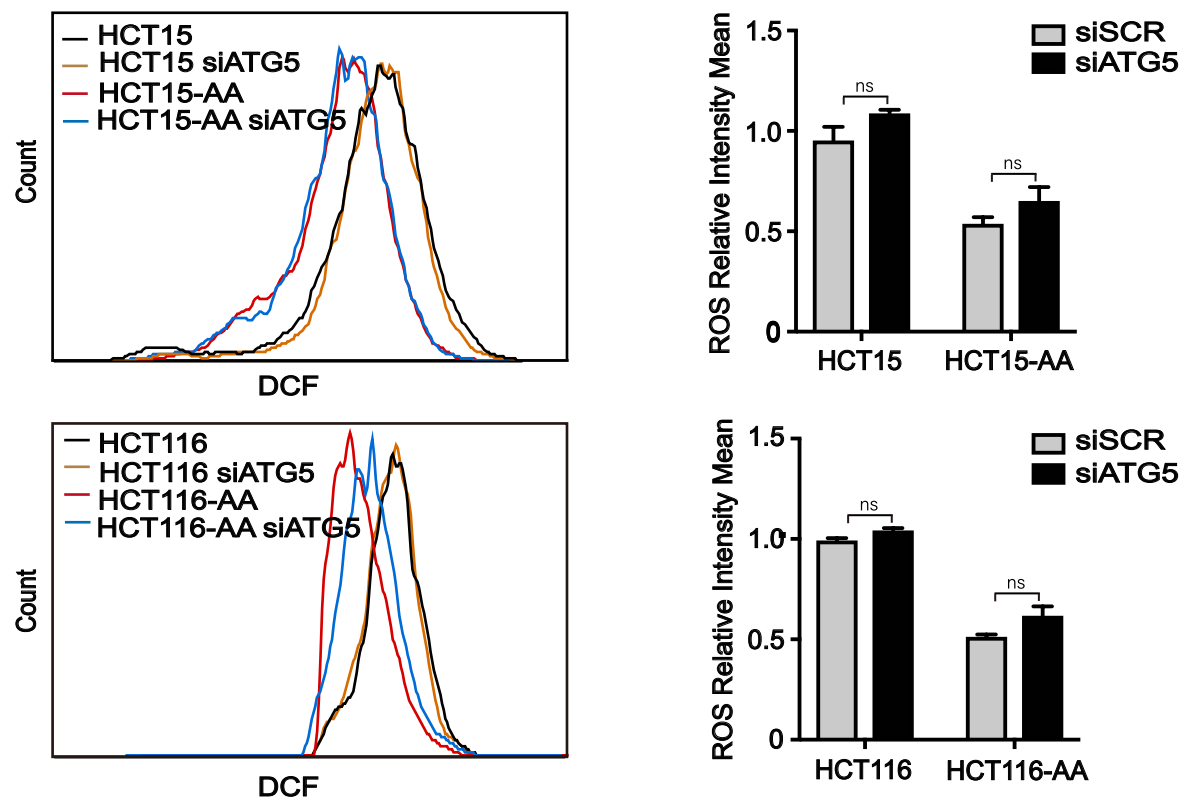

(b)

Supplement: Supplementary Materials — Figure S1: Autophagy flux was blocked by lysosomal inhibitor in CRC and CRC-AA cells. Figure S2: ER stress marker expressions in CRC and CRC-AA cells. Figure S3: The role of autophagy in reducing ROS in CRC-AA cells. Figure S4: CRC-AA cells are more sensitive to NF-κB inhibition or depletion. Figure S5: Upregulation of NF-κB is driven by GATA4 in CRC-AA cells. Figure S6: p62 depletion promotes CRC cell survival under acidic microenvironment. Figure S7: ICAM-1 expression in HCT116 cells. Table S1: Primers for RT quantitative PCR. Table S2: Protein array results. [file 8189485.f1.zip › Sup figure 3.pdf]

# Supplementary Figure 4

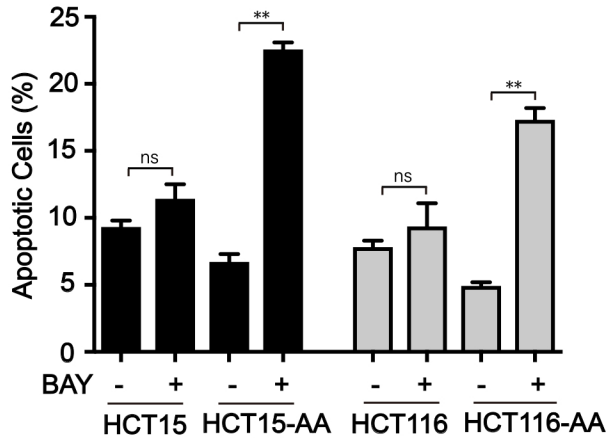

(a)

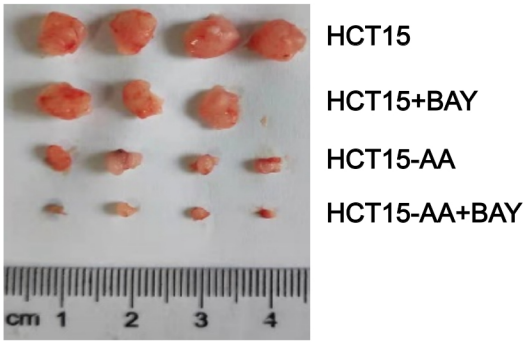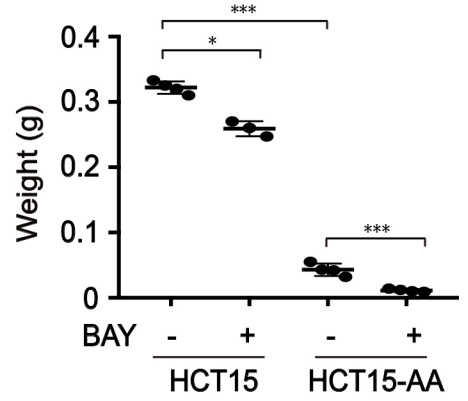

(b)

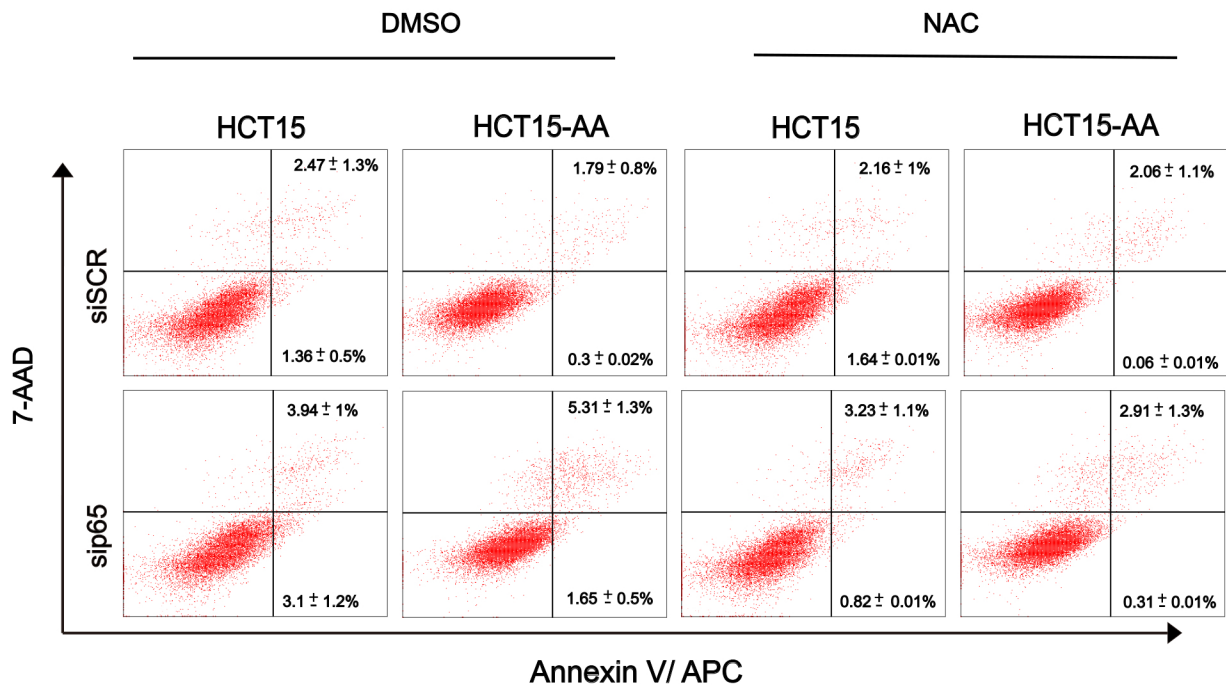

(c)

Supplement: Supplementary Materials — Figure S1: Autophagy flux was blocked by lysosomal inhibitor in CRC and CRC-AA cells. Figure S2: ER stress marker expressions in CRC and CRC-AA cells. Figure S3: The role of autophagy in reducing ROS in CRC-AA cells. Figure S4: CRC-AA cells are more sensitive to NF-κB inhibition or depletion. Figure S5: Upregulation of NF-κB is driven by GATA4 in CRC-AA cells. Figure S6: p62 depletion promotes CRC cell survival under acidic microenvironment. Figure S7: ICAM-1 expression in HCT116 cells. Table S1: Primers for RT quantitative PCR. Table S2: Protein array results. [file 8189485.f1.zip › sup figure 4.pdf]

## Supplementary Figure 5

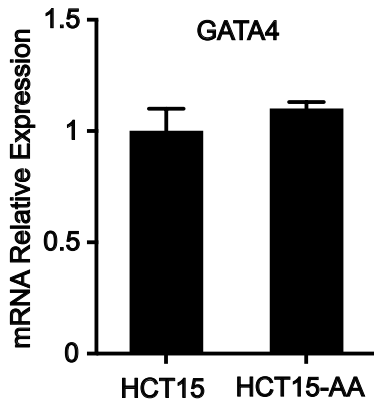

(a)

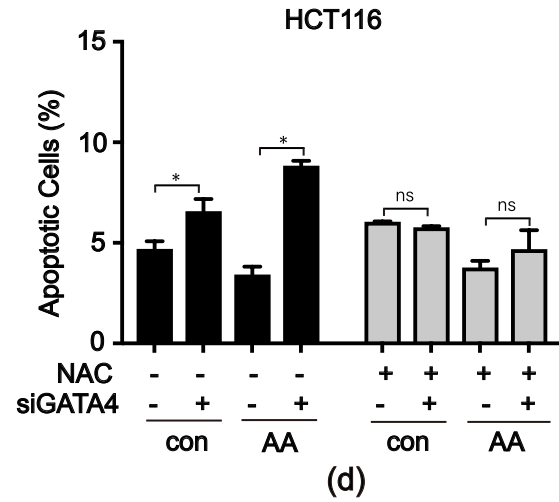

(d)

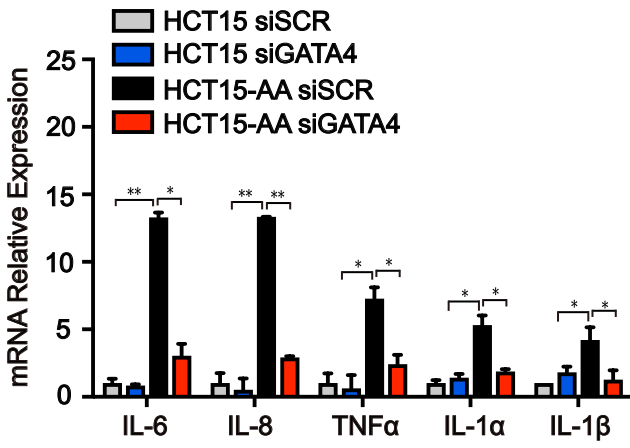

(b)

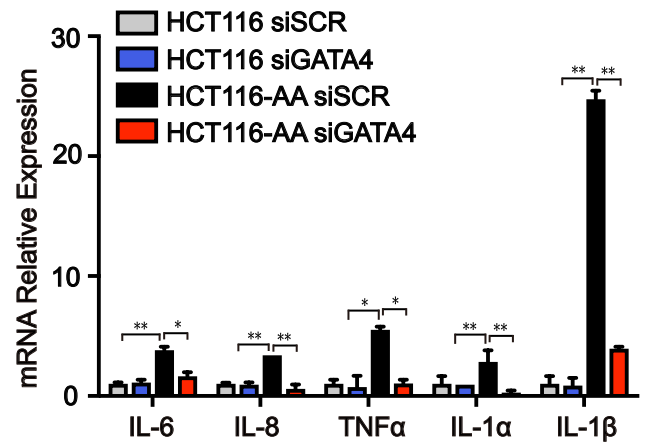

(c)

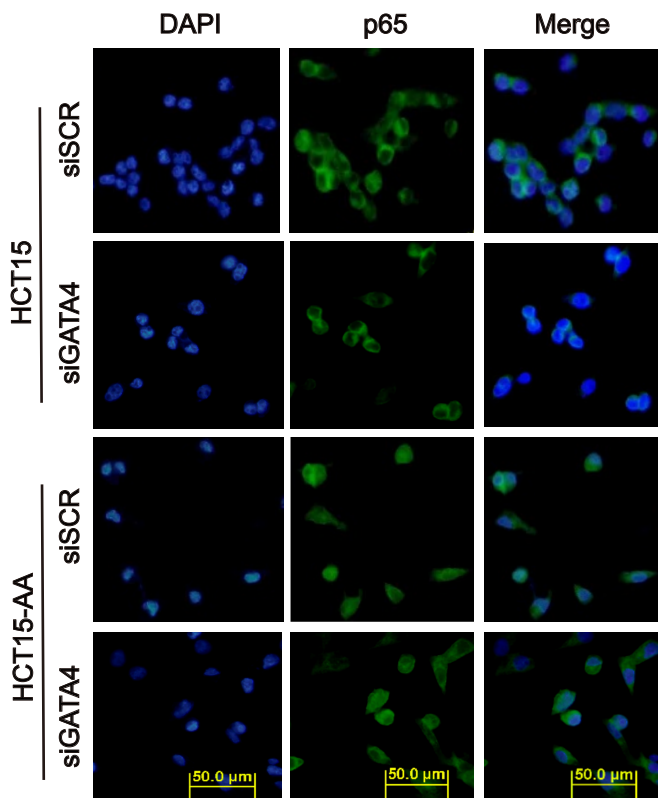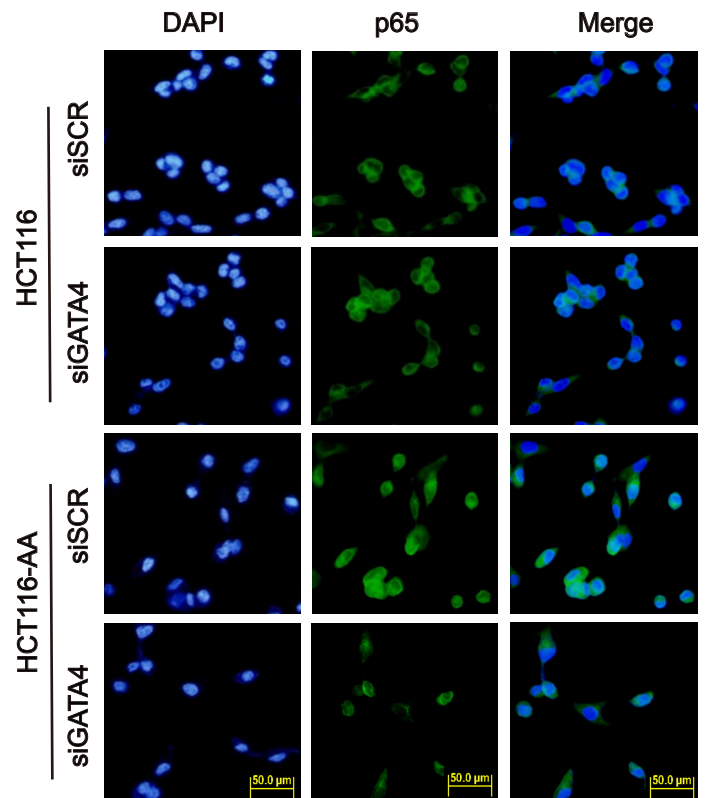

Supplement: Supplementary Materials — Figure S1: Autophagy flux was blocked by lysosomal inhibitor in CRC and CRC-AA cells. Figure S2: ER stress marker expressions in CRC and CRC-AA cells. Figure S3: The role of autophagy in reducing ROS in CRC-AA cells. Figure S4: CRC-AA cells are more sensitive to NF-κB inhibition or depletion. Figure S5: Upregulation of NF-κB is driven by GATA4 in CRC-AA cells. Figure S6: p62 depletion promotes CRC cell survival under acidic microenvironment. Figure S7: ICAM-1 expression in HCT116 cells. Table S1: Primers for RT quantitative PCR. Table S2: Protein array results. [file 8189485.f1.zip › sup figure 5.pdf]

Supplementary Figure 6

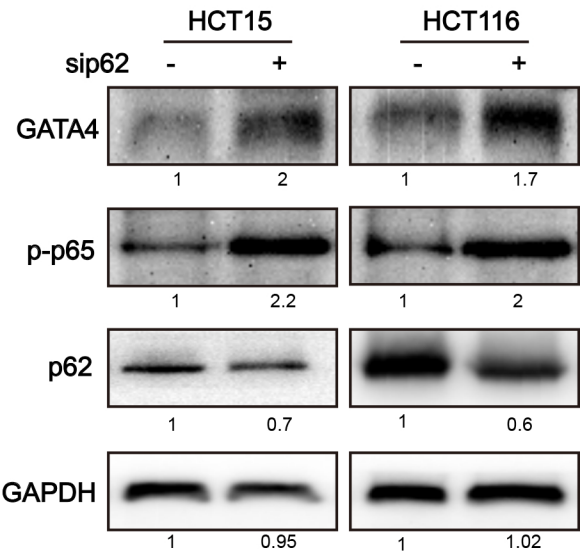

(a)

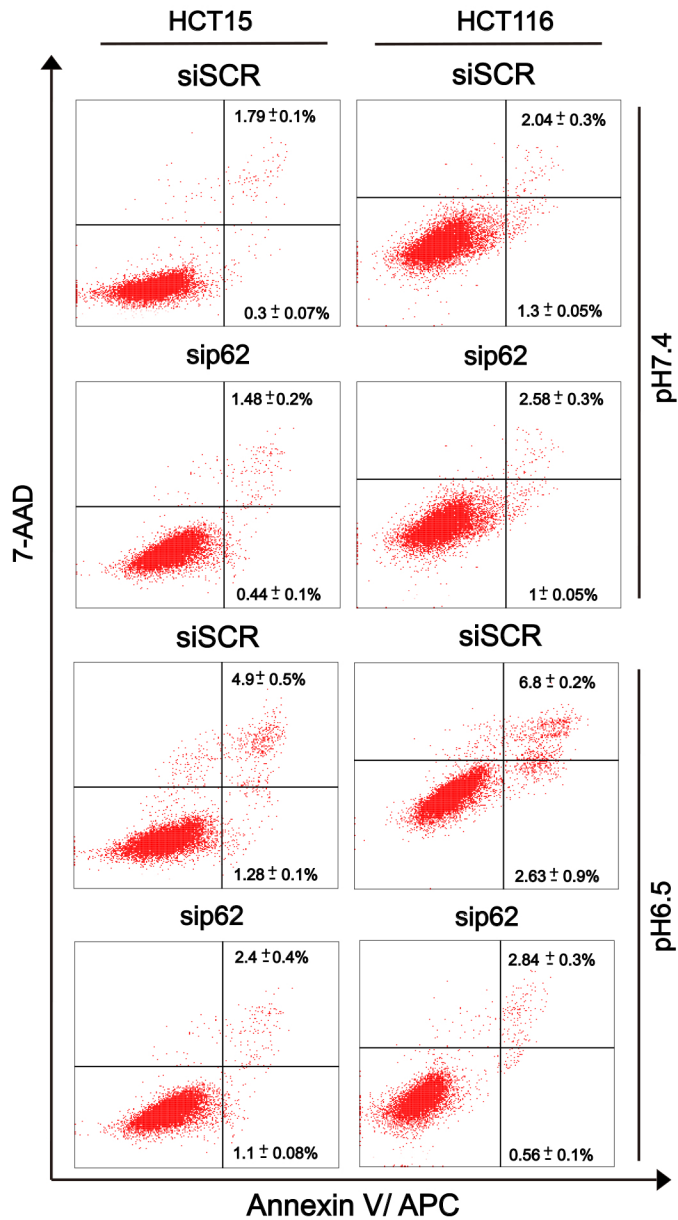

(b)

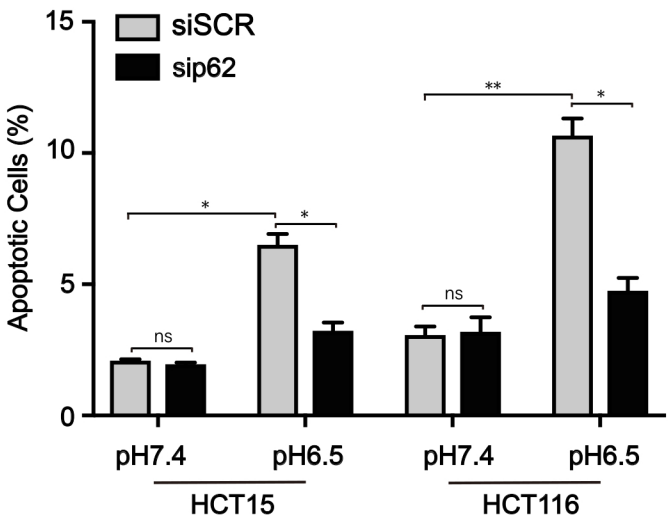

Supplement: Supplementary Materials — Figure S1: Autophagy flux was blocked by lysosomal inhibitor in CRC and CRC-AA cells. Figure S2: ER stress marker expressions in CRC and CRC-AA cells. Figure S3: The role of autophagy in reducing ROS in CRC-AA cells. Figure S4: CRC-AA cells are more sensitive to NF-κB inhibition or depletion. Figure S5: Upregulation of NF-κB is driven by GATA4 in CRC-AA cells. Figure S6: p62 depletion promotes CRC cell survival under acidic microenvironment. Figure S7: ICAM-1 expression in HCT116 cells. Table S1: Primers for RT quantitative PCR. Table S2: Protein array results. [file 8189485.f1.zip › sup figure 6.pdf]

Supplementary Figure 7

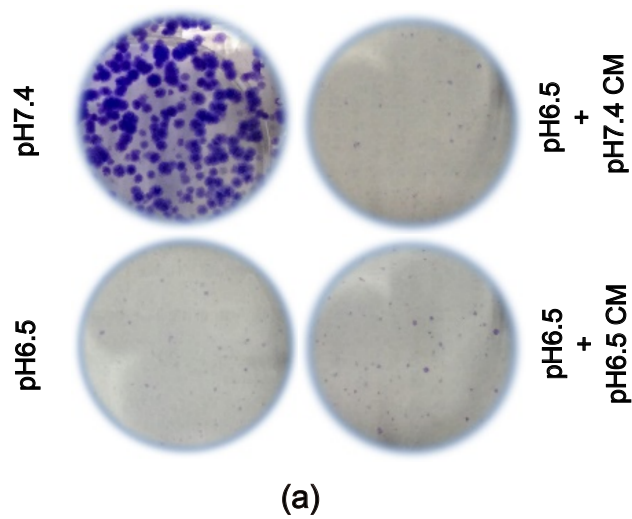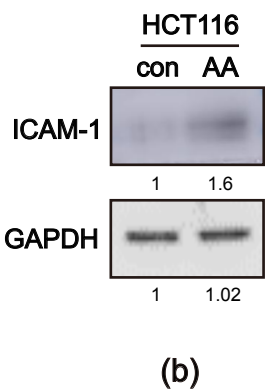

Supplement: Supplementary Materials — Figure S1: Autophagy flux was blocked by lysosomal inhibitor in CRC and CRC-AA cells. Figure S2: ER stress marker expressions in CRC and CRC-AA cells. Figure S3: The role of autophagy in reducing ROS in CRC-AA cells. Figure S4: CRC-AA cells are more sensitive to NF-κB inhibition or depletion. Figure S5: Upregulation of NF-κB is driven by GATA4 in CRC-AA cells. Figure S6: p62 depletion promotes CRC cell survival under acidic microenvironment. Figure S7: ICAM-1 expression in HCT116 cells. Table S1: Primers for RT quantitative PCR. Table S2: Protein array results. [file 8189485.f1.zip › sup figure 7.pdf]
